# Muscle Selection and Dosing in a Phase 3, Pivotal Study of AbobotulinumtoxinA Injection in Upper Limb Muscles in Children With Cerebral Palsy

**DOI:** 10.3389/fneur.2021.728615

**Published:** 2021-10-29

**Authors:** Joyce Oleszek, Ann Tilton, Jorge Carranza del Rio, Nigar Dursun, Marcin Bonikowski, Edward Dabrowski, Simon Page, Benjamin Regnault, Catherine Thompson, Mauricio R. Delgado

**Affiliations:** ^1^Department of Physical Medicine and Rehabilitation, University of Colorado and Children's Hospital Colorado, Aurora, IL, United States; ^2^LSUHSC and Children's Hospital New Orleans, New Orleans, LA, United States; ^3^Hospital San José Celaya, Celaya, Mexico; ^4^Department of Physical Medicine and Rehabilitation, Faculty of Medicine, Kocaeli University, Izmit, Turkey; ^5^Mazovian Neuropsychiatry Center, Warsaw, Poland; ^6^Beaumont Health, Oakland University School of Medicine, Grosse Pointe, MI, United States; ^7^Ipsen, Slough, United Kingdom; ^8^Consultant to Ipsen, Boulogne-Billancourt, France; ^9^June Pharma Consultant to Ipsen, Boulogne-Billancourt, France; ^10^Southwestern Medical Center, Scottish Rite Hospital for Children, University of Texas, Dallas, TX, United States

**Keywords:** abobotulinumtoxinA, botulinum toxin, cerebral palsy, dose, dosing, Dysport, spasticity, upper limb

## Abstract

**Background:** Guidelines recommend botulinum toxin-A in pediatric upper limb spasticity as part of routine practice. Appropriate dosing is a prerequisite for treatment success and it is important that injectors have an understanding on how to tailor dosing within a safe and effective range. We report upper limb dosing data from a phase 3 study of abobotulinumtoxinA injections in children with cerebral palsy.

**Methods:** This was a double-blind, repeat-treatment study (NCT02106351). In Cycle 1, children were randomized to abobotulinumtoxinA at 2 U/kg control dose or clinically relevant 8 U/kg or 16 U/kg doses. Doses were divided between the primary target muscle group (PTMG, wrist or elbow flexors) and additional muscles tailored to clinical presentation. During Cycles 2–4, children received doses of 8 U/kg or 16 U/kg and investigators could change the PTMG and other muscles to be injected. Injection of muscles in the other upper limb and lower limbs was also permitted in cycles 2–4, with the total body dose not to exceed 30 U/kg or 1,000 U (whichever was lower) in the case of upper and lower limb treatment.

**Results:** 212 children were randomized, of which 210 received ≥1 abobotulinumtoxinA injection. Per protocol, the elbow and wrist flexors were the most commonly injected upper limb muscles. Across all 4 cycles, the brachialis was injected in 89.5% of children (dose range 0.8–6 U/kg), the brachioradialis in 83.8% (0.4–3 U/kg), the flexor carpi ulnaris in 82.4% (0.5–3 U/kg) and the flexor carpi radialis in 79.5% (0.5–4 U/kg). Other frequently injected upper limb muscles were the pronator teres(70.0%, 0.3–3 U/kg). adductor pollicis (54.3%, 0.3-1 U/kg), pronator quadratus (44.8%, 0.1–2 U/kg), flexor digitorum superficialis (39.0%, 0.5-4 U/kg), flexor digitorum profundus (28.6%, 0.5–2 U), flexor pollicis brevis/opponens pollicis (27.6%, 0.3-1 U/kg) and biceps (27.1%, 0.5–6 U/kg). AbobotulinumtoxinA was well-tolerated at these doses; muscular weakness was reported in 4.3% of children in the 8 U/kg group and 5.7% in the 16 U/kg group.

**Conclusions:** These data provide information on the pattern of injected muscles and dose ranges used in this study, which were well-tolerated. Per protocol, most children received injections into the elbow and wrist flexors. However, there was a wide variety of other upper limb muscles injected as physicians tailored injection patterns to clinical need.

## Introduction

A majority of children with cerebral palsy (CP) have upper limb impairment that interferes with active and passive arm function leading to disability (1, 2). Depending on the underlying etiology and location of the brain abnormality, children may have various combinations of spasticity, weakness, dystonia, limited range of motion (ROM) and other positive and negative features of an upper motor neuron syndrome (3). Common patterns of upper limb involvement include elbow, wrist and finger flexion, thumb adduction, forearm pronation, and shoulder adduction-internal rotation (4, 5). Together, these features often contribute to difficulties in reaching, grasping, releasing, and manipulating objects, which can significantly impact function (2, 4, 6).

The cornerstone of spastic CP management is occupational therapy (OT) and/or physiotherapy, which is often combined with antispasticity pharmacotherapy in a long-term treatment program (7). Guidelines recommend botulinum toxin-A (BoNT-A) in pediatric upper limb (PUL) spasticity as part of routine practice (8) where injections are generally used to produce a selective reduction in muscle spasticity while optimizing the effects of therapies used for enhancing function and/or ease of care (7). In practice, chemodenervation with BoNT-A in the upper limb is mainly targeted to decrease flexor tone in the elbow, wrist, fingers and thumb, pronator tone in the forearm, or to decrease adductor and internal rotation tone in the shoulder.

We have previously reported the key efficacy and safety results from a large, double-blind, randomized phase 3 study of abobotulinumtoxinA (aboBoNT-A) for pediatric upper limb spasticity (9). Results from the first treatment cycle showed that aboBoNT-A at doses of 8 U/kg and 16 U/kg were well tolerated and demonstrated significant improvements in muscle tone vs. a low-dose 2 U/kg control. Efficacy was sustained with repeated treatment (up to 4 cycles) and the majority of children achieved their treatment goals at least as expected (9). Both doses were well tolerated, and the study formed the basis for regulatory approval of aboBoNT-A in several regions.

Until now, evidence-based information on muscle selection and BoNT-A (including aboBoNT-A) dosing for clinical use in the pediatric upper limb has been sparse and mainly based on small studies and expert opinion (10, 11). However, appropriate dosing is a prerequisite for treatment success and it is important that clinicians have an understanding on how to tailor dosing within a safe and efficacious range. This paper presents the results of an analysis of dosing from the phase 3 study of aboBoNT-A in pediatric upper limb spasticity and aims to provide a detailed description of injection parameters, within the context of a double-blind study.

## Methods

### Study Conduct and Participants

The Dysport in PUL spasticity study (NCT02106351) was a double-blind, repeat treatment (up to 4 cycles) pivotal trial, methodological details of which have been previously published (9). Institutional review boards at the 32 participating sites (across Belgium, the Czech Republic, Poland, Spain, Turkey, Israel, Mexico, and the USA) approved the protocol, and the trial was executed in accordance with the Declaration of Helsinki and International Conference on Harmonization Good Clinical Practice Guidelines.

In brief, this multicenter study included children (aged 2–17 years, weighing ≥ 10 kg) with a diagnosis of CP (12) and spasticity in at least one upper limb. Children were eligible for inclusion if they had a Modified Ashworth Scale (MAS) score ≥ 2 in the primary targeted muscle group (PTMG; elbow or wrist flexors). Children with a fixed contracture in the PTMG (defined for this study as <40° of available ROM at elbow or wrist joint) were excluded from this study as were children with choreoathetoid/dystonic movements, history of aspiration or dysphagia, previous/planned surgery of the PTMG, and phenol/alcohol injections within the past year.

### Study Treatment

In the first cycle, children were randomized to aboBoNT-A 2, 8 or 16 U/kg into the designated study upper limb using electrical stimulation and/or ultrasound to localize the targeted injection sites. The dose for each child was calculated according to their body weight, up to a maximum body weight of 40 kg (even if the child weighed more than 40 kg, and therefore, a maximum total dose of 80 U in the control group, 320 U in the 8 U/kg group and 640 U in the 16 U/kg group). If elbow flexors were chosen as the PTMG, both the brachialis and brachioradialis had to be injected; if the wrist flexors were chosen as the PTMG, both the flexor carpi radialis and flexor carpi ulnaris had to be injected. Other muscles in the study limb were injected based on clinical presentation and the individualized treatment goals. To maintain blinding across the treatment groups a fixed volume of 1.6 mL was injected. The PTMG was injected with a pre-defined volume (elbow flexors: brachialis 0.6 mL, brachioradialis 0.3 mL; wrist flexors: flexor carpi radialis 0.4 mL, flexor carpi ulnaris 0.3 mL) and maximum volumes were defined for the other upper limb muscles (Supplementary Table 1). In addition to any existing physiotherapy or occupational therapy (which was to remain stable throughout the study), all children were to participate in a personalized, goal-oriented home exercise therapy program [HETP, minimum of 5 x 15-min sessions per week (13)] to provide a standardized background of good practice after BoNT-A therapy.

During Cycles 2–4, the dose remained blinded and children received doses of 8 U/kg or 16 U/kg in the study limb, with allocation of the patients who previously received 2 U/kg to one of these treatment groups, except if changes were clinically necessary to manage efficacy/tolerability (minimum 2 U/kg, maximum 16 U/kg). Eligibility for retreatment was individualized as determined by clinical need based on pre-defined criteria and was assessed from week 16 onwards (9). Investigators could change between wrist and elbow flexors as the PTMG and could also modify other muscles to be injected based on clinical need. The total volume in the study limb remained 1.6 mL, with the aforementioned volume reserved for injection in PTMG. In addition, treatment of other limbs could occur, if deemed clinically necessary, with injection of muscles in the other upper limb (up to 5 U/kg or 200 U) and lower limbs (up to 10 U/kg or 360 U) permitted, with the total body dose not to exceed 30 U/kg or 1,000 U, whichever was lower, when both upper and lower limb treatment were combined).

### Analysis

Descriptive statistics (mean, standard deviation [SD], range, percentages) were used to characterize demographics, AboBoNT-A dosage and safety data (treatment-emergent adverse events, TEAEs) for all treated children. Analyses of muscle injection frequency included all children in all cycles (i.e., including 2 U/kg in Cycle 1). AboBoNT-A dosing was analyzed by the individual muscles treated, regardless of whether they were selected as PTMG; dose ranges mean (minimum-maximum) are presented across all 4 cycles for children treated with the clinically relevant doses of 8 U/kg and 16 U/kg groups. TEAEs were monitored by direct, non-leading questioning or by spontaneous reports and were analyzed by doses given in the study upper limb and by total body dose.

## Results

### Patient Flow and Baseline Characteristics

**Figure 1:**
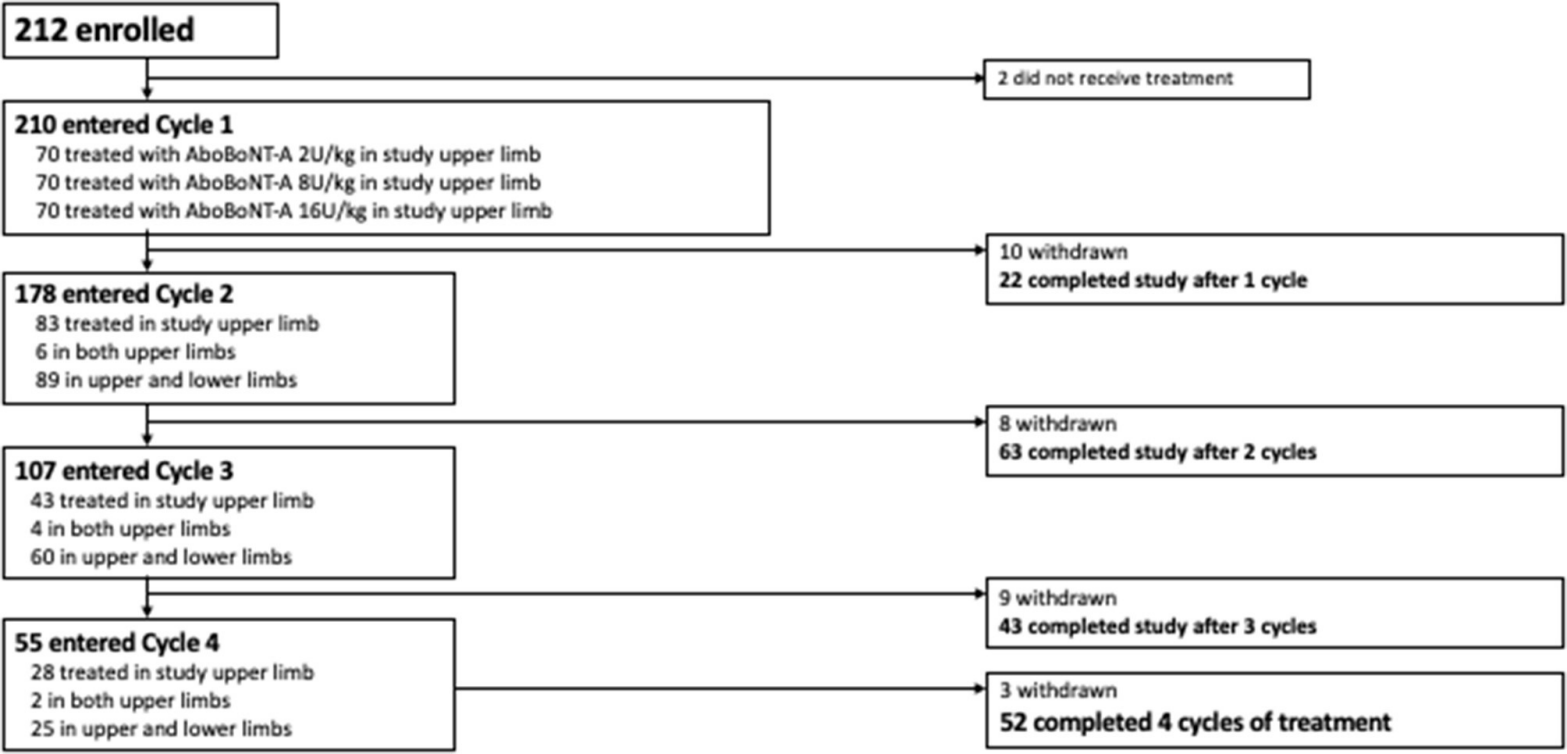
Patient disposition. AboBoNT-A, abobotulinumtoxinA.

Of the 226 children screened, 212 were randomized to treatment and 210 received ≥ 1 aboBoNT-A injection and were included in this analysis. Baseline characteristics for the overall population are provided in Table 1. Overall, 56.7% of children were aged 2–9 years and 76.9% had hemiparesis. A total of 210 children entered Cycle 1, 178 entered Cycle 2, 107 entered Cycle 3 and 55 entered Cycle 4 (Figure 1). The mean time to retreatment was 24.7 weeks following the cycle 1 injection (8 U/kg and 16 U/kg groups combined), 19.4 following the cycle 2 injection and 17.4 weeks following the cycle 3 injection (9).

**Table 1:** Baseline characteristics.

**Parameter**	**AboBoNT-A *N* = 210**
Age (years); mean ± SD 2–9 Years, *n* (%) 10–17 Years, *n* (%)	9.0 ± 4.4 120 (57.1%) 90 (42.9%)
**Sex**, ***n*** **(%)**	
Male Female	126 (60.0%) 84 (40.0%)
Weight, (kg); mean ± SD	32.2 ± 16.9
**Pattern of paresis**	
Unilateral Bilateral Other	160 (76.9%) 45 (21.6%) 3 (1.4%)
**GMFCS level**, ***n*** **(%)**	
I II III IV	95 (45.2%) 63 (30.0%) 11 (5.2%) 41 (19.5%)
**MAS; mean** **±** **SD**	
PTMG Elbow Wrist	3.1± 0.4 2.8 ± 0.8 2.5 ± 1.1
Prior BoNT-A treatment, *n* (%)	138 (66.3%)
Concomitant medications^a^, *n* (%) Baclofen Antiepileptics Trihexiphenidyl Clonazepam Diazepam	*N* = 208 18 (8.7%) 3 (1.4%) 2 (1.0%) 1 (0.5%) 1 (0.5%)

*AboBoNT-A, abobotulinumtoxinA; GMFCS, gross motor function classification system; MAS, modified Ashworth scale; PTMG, primary target muscle group*.

a *reported by investigator as medications for spasticity*.

### AbobotulinumtoxinA Dosing and Commonly Injected Muscles

**Figure 2:**
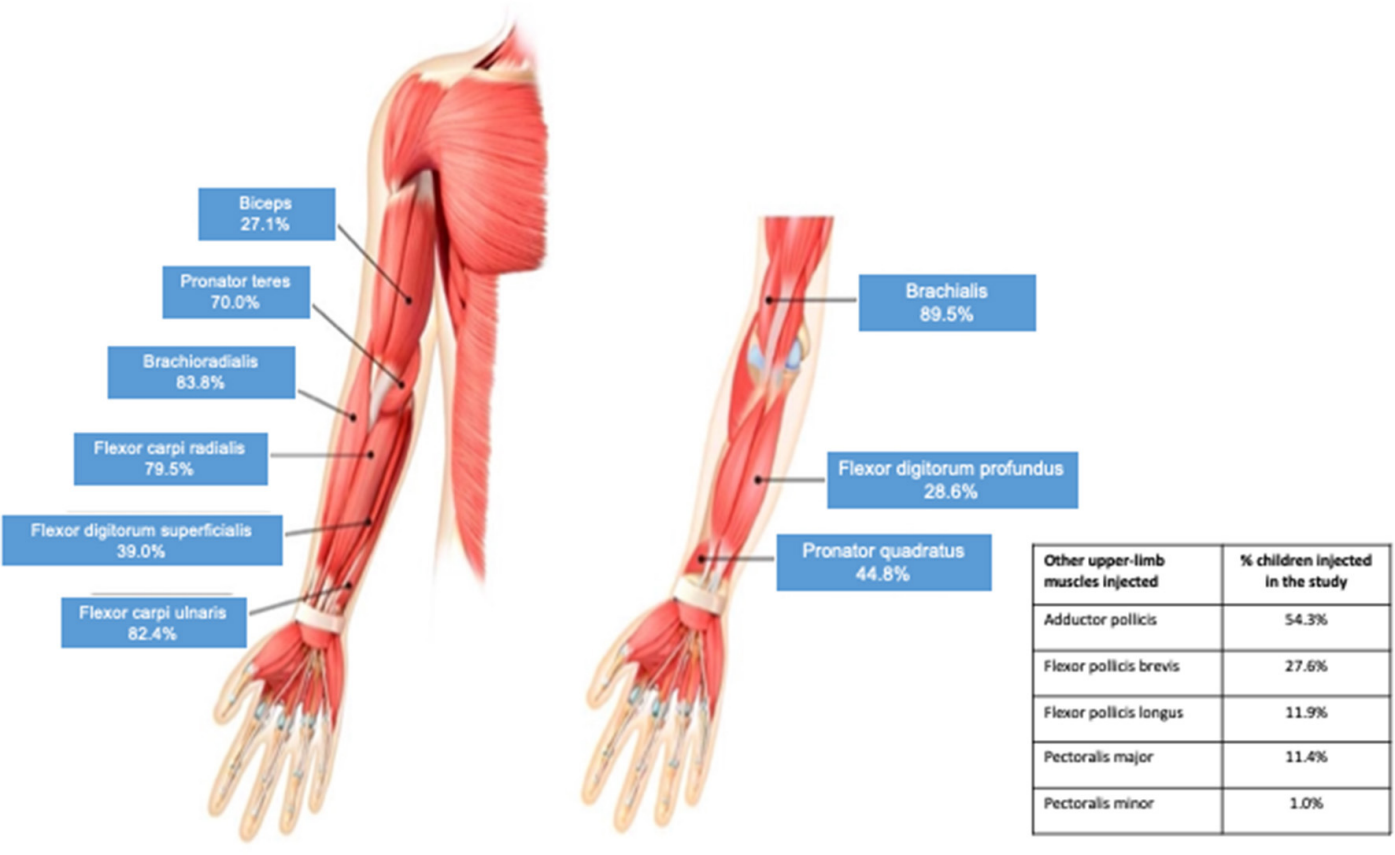
Muscles most commonly injected for pediatric upper limb spasticity (all cycles).

In line with the study protocol, the elbow and wrist flexors were the most commonly injected upper limb muscles (Figure 2). Across all four cycles, the brachialis was injected in 89.5% of children, the brachioradialis in 83.8%, the flexor carpi ulnaris in 82.4% and the flexor carpi radialis in 79.5%. The next most frequently injected muscle was the pronator teres, which was targeted in 70.0% of children. Other frequently injected upper limb muscles were the biceps, flexor digitorum superficialis, flexor digitorum profundus, pronator quadratus, adductor pollicis, flexor pollicis brevis and opponens pollicis. As shown in Table 2, the frequency of injection per muscle remained broadly consistent across the four treatment cycles. Table 2 also provides descriptive dosing data for children treated with 8 U/kg and 16 U/kg across the treatment cycles.

**Table 2:** Mean [Range] abobotulinumtoxinA doses per upper limb muscle across treatment cycles.

	**Cycle 1**		**Cycle 2**		**Cycle 3**		**Cycle 4**	
	**AboBoNT-A** **8 U/kg (*N* = 70)**	**AboBoNT-A 16 U/kg (*N* = 70)**	**AboBoNT-A 8 U/kg (*N* = 86)**	**AboBoNT-A 16 U/kg (*N* = 90)**	**AboBoNT-A 8 U/kg (*N* = 45)**	**AboBoNT-A 16 U/kg (*N* = 57)**	**AboBoNT-A 8 U/kg (*N* = 20)**	**AboBoNT-A 16 U/kg (*N* = 33)**
**Brachialis**, ***n*** **(%)**	61 (87.1%)	57 (81.4%)	76 (88.4%)	74 (82.2%)	40 (88.9%)	50 (87.7%)	18 (90.0%)	30 (90.9%)
Dose (U/kg); Mean [Range]	2.75 [1.0–3.0]	5.32 [2.0–6.0]	2.76 [1.0–3.0]	5.31 [2.0–6.0]	2.85 [1.0–3.0]	5.38 [2.0–6.0]	2.92 [1.5–3.0]	5.30 [2.0–6.0]
**Brachioradialis**, ***n*** **(%)**	59 (84.3%)	54 (77.1%)	72 (83.7%)	68 (75.6%)	39 (86.7%)	42 (73.7%)	18 (90.0%)	24 (72.7%)
Dose (U/kg); Mean [Range]	1.42 [0.5–1.5]	2.75 [1.0–3.0]	1.42 [0.5–1.5]	2.78 [1.0–3.0]	1.46 [1.0–1.5]	2.74 [1.0–3.0]	1.50 [1.5–1.5]	2.88 [2.0–3.0]
**Flexor carpi ulnaris**, ***n*** **(%)**	54 (77.1%)	58 (82.9%)	63 (73.3%)	69 (76.7%)	30 (66.7%)	41 (71.9%)	15 (75.0%)	25 (75.8%)
Dose (U/kg); Mean [Range]	1.44 [0.5–1.5]	2.84 [1.0–3.0]	1.36 [0.5–1.5]	2.81 [1.0–3.0]	1.48 [1.0–1.5]	2.85 [1.0–3.0]	1.50 [1.5–1.5]	2.76 [1.0–3.0]
**Flexor carpi radialis**, ***n*** **(%)**	50 (71.4%)	56 (80.0%)	58 (67.4%)	66 (73.3%)	28 (62.2%)	37 (64.9%)	15 (75.0%)	23 (69.7%)
Dose (U/kg); Mean [Range]	1.87 [0.5–2.0]	3.75 [1.0–4.0]	1.91 [1.0–2.0]	3.56 [1.0–4.0]	1.88 [0.5–2.0]	3.57 [1.0–4.0]	1.90 [0.5–2.0]	3.48 [1.0–4.0]
**Pronator teres**, ***n*** **(%)**	39 (55.7%)	44 (62.9%)	48 (55.8%)	52 (57.8%)	20 (44.4%)	32 (56.1%)	5 (25.0%)	12 (36.4%)
Dose (U/kg); Mean [Range]	0.99 [0.5–1.0]	1.98 [1.0–3.0]	1.00 [0.5–1.5]	1.94 [1.0–2.0]	0.95 [0.5–1.0]	1.97 [1.0–2.0]	0.90 [0.5–1.0]	1.92 [1.0–2.0]
**Pronator quadratus**, ***n*** **(%)**	20 (28.6%)	24 (34.3%)	23 (26.7%)	37 (41.1%)	10 (22.2%)	20 (35.1%)	4 (20.0%)	12 (36.4%)
Dose (U/kg); Mean [Range]	0.50 [0.5–0.5]	1.00 [1.0–1.0]	0.50 [0.5–0.5]	1.00 [1.0–1.0]	0.50 [0.5–0.5]	1.05 [1.0–2.0]	0.50 [0.5–0.5]	1.00 [1.0–1.0]
**Biceps**, ***n*** **(%)**	9 (12.9%)	11 (15.7%)	15 (17.4%)	18 (20.0%)	11 (24.4%)	14 (24.6%)	2 (10.0%)	8 (24.2%)
Dose (U/kg); Mean [Range]	2.11 [0.5–3.0)	4.18 [2.0–6.0]	2.17 [1.0–3.0]	4.11 [1.0–6.0]	2.27 [1.5–3.0]	4.71 [1.0–6.0]	2.25 [2.0–2.5]	4.38 [2.0–6.0]
**Adductor pollicis**, ***n*** **(%)**	21 (30.0%)	28 (40.0%)	28 (32.6%)	40 (44.4%)	14 (31.1%)	27 (47.4%)	7 (35.0%)	15 (45.5%)
Dose (U/kg); Mean [Range]	0.50 [0.5–0.5]	1.00 [1.0–1.0]	0.50 [0.5–0.5]	1.00 [1.0–1.0]	0.50 [0.5–0.5]	1.00 [1.0–1.0]	0.50 [0.5–0.5]	1.00 [1.0–1.0]
**Flexor digitorum superficialis**, ***n*** **(%)**	21 (30.0%)	17 (24.3%)	27 (31.4%)	28 (31.1%)	12 (26.7%)	18 (31.6%)	6 (30.0%)	11 (33.3%)
Dose (U/kg);Mean [Range]	1.38 [1.0–1.5]	2.71 [2.0–3.0]	1.31 [1.0–1.5]	2.68 [1.0–4.0]	1.33 [1.0–1.5]	2.67 [1.0–3.0]	1.25 [1.0–1.5]	2.45 [1.0–3.0]
**Flexor digitorum profundus, n (%)**	14 (20.0%)	13 (18.6%)	15 (17.4%)	16 (17.8%)	9 (20.0%)	11 (19.3%)	4 (20.0%)	5 (15.2%)
Dose (U/kg); Mean [Range]	0.96 [0.5–1.0]	1.85 [1.0–2.0]	0.97 [0.5–1.0]	1.94 [1.0–2.0]	1.00 [1.0–1.0]	1.73 [1.0–2.0]	1.00 [1.0–1.0]	1.80 [1.0–2.0]
**Flexor pollicis brevis opponens**	11 (15.7%)	16 (22.9%)	13 (15.1%)	22 (24.4%)	3 (6.7%)	14 (24.6%)	2 (10.0%)	7 (21.2%)
**pollicis**, ***n*** **(%)**								
Dose (U/kg); Mean [Range]	0.50 [0.5–0.5]	1.00 [1.0–1.0]	0.50 [0.5–0.5]	1.00 [1.0–1.0]	0.50 [0.5–0.5]	1.00 [1.0–1.0]	0.50 [0.5–0.5]	1.00 [1.0–1.0]
**Flexor pollicis longus**, ***n*** **(%)**	3 (4.3%)	1 (1.4%)	8 (9.3%)	9 (10.0%)	3 (6.7%)	2 (3.5%)	2 (10.0%)	2 (6.1%)
Dose (U/kg); Mean [Range]	0.83 [0.5–1.0]	1.00 [1.0–1.0]	0.75 [0.5–1.0]	1.11 [1.0–2.0]	0.83 [0.5–1.0]	1.00 [1.0–1.0]	0.75 [0.5–1.0]	2.00 [2.0–2.0]
**Pectoralis major, n (%)**	4 (5.7%)	4 (5.7%)	6 (7.0%)	9 (10.0%)	3 (6.7%)	4 (7.0%)	1 (5.0%)	4 (12.1%)
Dose (U/kg); Mean [Range]	1.88 [1.0–2.5]	4.00 [2.0–5.0]	1.92 [1.0–2.5]	4.00 [1.0–5.0]	2.00 [1.0–2.5]	4.50 [4.0–5.0]	1.00 [1.0–1.0]	4.50 [3.0–5.0]
**Pectoralis minor, n (%)**	0	1 (1.4%)	0	1 (1.1%)	0	0	0	0
Dose (U/kg); Mean [Range]	–	2.00 [2.0–2.0]	–	1.00 [1.0–1.0]	–	–	–	–

*AboBoNT-A, abobotulinumtoxinA; U, units*.

From Cycle 2 onwards, over half of children (59.9%) also received at least one injection into the lower limbs. Within each treatment cycle, a relatively consistent pattern was observed with the proportion of patients receiving treatment in lower limbs approaching 50% in each cycle (Table 3). The most frequently injected lower limb muscles were the gastrocnemius/soleus/tibialis posterior (injected in 45.5% of children), hamstrings (20.8%) and the hip adductors (7.9%).

**Table 3:** Proportion of children receiving injections into other (non-study) limbs.

	**Number (%) of children**						
**Where treated**	**Cycle 2**		**Cycle 3**		**Cycle 4**		**Cycles 2 to 4**
	**8 U/kg**	**16 U/kg**	**8 U/kg**	**16 U/kg**	**8 U/kg**	**16 U/kg**	**Any dose^a^**
	***N* = 86**	**(*N* = 90)**	**(*N* = 45)**	**(*N* = 57)**	**(*N* = 20)**	**(*N* = 33)**	**(*N* = 178)**
Study upper limb only	40 (46.5%)	42 (46.7%)	20 (44.4%)	21 (36.8%)	14 (70.0%)	13 (39.4%)	70 (39.3%)
Both upper limbs	2 (2.3%)	3 (3.3%)	1 (2.2%)	2 (3.5%)	0	2 (6.1%)	7 (3.9%)
Study upper limb and lower limb	43 (50.0%)	44 (48.9%)	23 (51.1%)	33 (57.9%)	6 (30.0%)	18 (54.5%)	100 (56.2%)
Both upper limbs and the lower limbs	1 (1.2%)	1 (1.1%)	1 (2.2%)	1 (1.8%)	0	0	3 (1.7%)

a *Includes children who had dose reductions to 2 U/kg or 4 U/kg*.

The majority of children did not have a change in dose (except for the per-protocol randomized switch from low-dose control in Cycle 1 to either 8 U/kg or 16 U/kg in Cycle 2 onwards). Across all cycles, there were 11 children who received a dose decrease and 9 children who received a dose increase based on clinical discretion; the investigator remained blinded to dose throughout.

### Safety of AbobotulinumtoxinA Dosing in Children With Upper Limb Spasticity

As previously reported, aboBoNT-A was well tolerated across treatment cycles and the incidence of TEAEs tended to decrease across the four treatment cycles (9). Similar to previous studies of aboBoNT-A for lower limb spasticity in a similar population of children (14, 15), the most frequently reported TEAEs were related to common childhood infections (comprised of upper respiratory tract infections, pharyngitis, sinusitis and urinary tract infections) and were considered unrelated to study drug (Table 4). The incidence of TEAEs assessed as related to treatment by the investigator was low across all treatment cycles in Cycle 1 (8.6% children each in aboBoNT-A 8 U/kg and 16 U/kg group), and did not increase across the treatment cycles (7.0, 2.2, 5.0% of children in the 8 U/kg group and 6.7, 3.5, 0% in the 16 U/kg group in cycles 2, 3, 4, respectively).

**Table 4:** Treatment emergent adverse events.

	**Cycle 1**		**Cycle 2**		**Cycle 3**		**Cycle 4**	
	**8 U/kg (*N* = 70)**	**16 U/kg (*N* = 70)**	**8 U/kg (*N* = 86)**	**16 U/kg (*N* = 90)**	**8 U/kg (*N* = 45)**	**16 U/kg (*N*=57)**	**8 U/kg (*N* = 20)**	**16 U/kg (*N* = 33)**
**Any TEAE** *Infections and infestations* *Gastrointestinal disorders* *Nervous system disorders* *Respiratory, thoracic and mediastinal disorders* *General disorders and administration site conditions* *Injury, poisoning and procedural complications* *Musculoskeletal and connective tissue disorders* *Skin and subcutaneous tissue disorders*	40 (57.1) 23 (32.9) 8 (11.4) 7 (10.0) 7 (10.0) 6 (8.6) 6 (8.6) 6 (8.6) 6 (8.6)	33 (47.1) 21 (30.0) 5 (7.1) 4 (5.7) 3 (4.3) 3 (4.3) 2 (2.9) 6 (8.6) 2 (2.9)	41 (47.7) 21 (24.4) 3 (3.5) 5 (5.8) 4 (4.7) 13 (15.1) 6 (7.0) 4 (4.7) 5 (5.8)	28 (31.1) 19 (21.1) 0 4 (4.4) 3 (3.3) 3 (3.3) 4 (4.4) 6 (6.7) 2 (2.2)	20 (44.4) 15 (33.3) 2 (4.4) 3 (6.7) 2 (4.4) 1 (2.2) 3 (6.7) 3 (6.7) 3 (6.7)	19 (33.3) 9 (15.8) 4 (7.0) 4 (7.0) 5 (8.8) 2 (3.5) 3 (5.3) 4 (7.0) 2 (3.5)	13 (65.0) 6 (30.0) 1 (5.0) 3 (15.0) 2 (10.0) 2 (10.0) 1 (5.0) 1 (5.0) 1 (5.0)	9 (27.3) 6 (18.2) 0 0 1 (3.0) 0 1 (3.0) 0 2 (6.1)
**TEAEs judged as potentially related to study treatment**	6 (8.6)	6 (8.6)	6 (7.0)	6 (6.7)	1 (2.2)	2 (3.5)	1 (5.0)	0
**Musculoskeletal and connective tissue disorders** *Muscular weakness* *Arthralgia* *Myalgia*	3 (4.3) 3 (4.3) 0 0	5 (7.1) 4 (5.7) 0 1 (1.4)	0 0 0 0	5 (5.6) 5 (5.6) 0 0	1 (2.2) 1 (2.2) 0 0	2 (3.5) 1 (1.8) 1 (1.8) 0	0 0 0 0	0 0 0 0
**Gastrointestinal disorders** *Nausea* *Salivary hypersecretion* *Vomiting*	2 (2.9) 1 (1.4) 1 (1.4) 0	0 0 0 0	1 (1.2) 0 0 1 (1.2)	0 0 0 0	0 0 0 0	0 0 0 0	0 0 0 0	0 0 0 0
**Nervous system disorders** *Headache* *Seizure* *Balance disorder*	2 (2.9) 1 (1.4) 1 (1.4) 0	0 0 0 0	1 (1.2) 0 1 (1.2) 0	1 (1.1) 0 0 1 (1.1)	0 0 0 0	0 0 0 0	0 0 0 0	0 0 0 0
**General disorders and administration site conditions** *Asthenia* *Fatigue* *Injection site bruising* *Injection site pain* *Injection site rash* *Pyrexia*	1 (1.4) 1 (1.4) 0 0 0 0 0	1 (1.4) 0 0 0 0 0 1 (1.4)	3 (3.5) 0 1 (1.2) 0 1 (1.2) 1 (1.2) 0	1 (1.1) 0 0 0 1 (1.1) 0 0	0 0 0 0 0 0 0	0 0 0 0 0 0 0	1 (5.0) 0 0 1 (5.0) 0 1 (5.0) 0	0 0 0 0 0 0 0
**Skin and subcutaneous tissue disorders** *Hyperhidrosis*	0 0	0 0	1 (1.2) 1 (1.2)	0 0	0 0	0 0	0 0	0 0

*TEAE, treatment emergent adverse event*.

TEAEs of muscular weakness were localized in all but one case, mild or moderate in severity and occurred within the first 4 weeks post-injection and all resolved with highly variable durations (14 to 234 days). Muscular weakness across treatment cycles (Cycles 1, 2, and 3, respectively) occurred in a total of 3 (4.3%), 0 (0%) and 1 (2.2%) children in the 8 U/kg group, and 4 (5.7%), 5 (5.6%) and 1 (1.8%) children in the 16 U/kg group. Muscular weakness was not reported in either group in Cycle 4. Of the children who had local muscular weakness and repeat treatment, three had a dose reduction (from 16 U/kg to 8 U/kg). One child had repeat local muscular weakness at the decreased dose and the other two did not experience repeat events. Another child had localized muscular weakness over two consecutive cycles at the 16 U/kg dose. One child treated with aboBoNT-A 8 U/kg reported a severe case of local muscular weakness in the hand. The child subsequently discontinued from the study due to need for further BoNT-A treatment–but not in the study upper limb. One child with tetraparesis (GMFCS Level III) treated with aboBoNT-A 8 U/kg experienced a non-serious TEAE of generalized muscular weakness, starting from Day 8 post-injection in Cycle 1 and lasting 22 days. The child continued with treatment in Cycles 2 and 3 (including lower limb injections to a total dose of 18 U/kg) without any further event of muscular weakness.

Overall, there were no clinically relevant differences in the frequency or severity of the TEAEs reported when considering total body doses from Cycle 2 onwards (i.e., when the maximum permitted total body aboBoNT-A dose for any treatment cycle was 30 U/kg or 1,000 U in case of concurrent treatment of both upper and lower limbs). When divided into total body dosing categories, 25–31% of children were treated with aboBoNT-A 10 U/kg between Cycles 2 and 4, 55–59% with 20 U/kg, and 12–18% with 30 U/kg. The overall incidence of TEAEs did not increase with increasing total body dose administered being 44.4–58.8%, 37.9–42.4% and 12.5–31.8% in the 10 U/kg, 20 U/kg and 30 U/kg total body dose group categories, respectively. No single TEAE was reported in >2 children in the total body dose group of 30 U/kg and there were no reports of treatment-related TEAE or serious TEAE in this highest dose category.

## Discussion

These data provide information on the muscles selected and the dose ranges used during this phase 3 study, which were well tolerated and shown previously to be effective in improving spasticity (9). In line with the protocol, most children received injections into the elbow and wrist flexors. However, a wide variety of other upper limb muscles were also injected, as physicians tailored injection patterns to individual patients. Once permitted by the study protocol, half (50%) of children also received simultaneous injections into the lower limb(s).

In typical hemiplegic posturing, which was the most common presentation in this study, the most common upper limb target muscles previously reported were the brachialis, pronator teres, flexor carpi ulnaris, flexor carpi radialis and the adductor pollicis (16), and the most commonly injected muscles observed in our study (outside of the PTMG) align with this pattern. However, about a quarter of children also received injections into the fingers and thumb flexors highlighting the need to treat the hand. For example, opening the hand with correct wrist position is considered essential for function; individual goal examples might include managing to grasp a school lunch tray (active goal) or to improve ease of hand splint wearing (passive goal). In addition, about one in ten children received an injection into the shoulder muscles aiming for an improvement in reaching tasks, which might include activities such as hair brushing (active goal), or facilitation of dressing and undressing by the caregiver (passive goals).

In our study, whilst the 8 U/kg and 16 U/kg doses produced a statistically significant greater effect on muscle tone compared to 2 U/kg, we observed that children in all groups (2 U/kg, 8 U/kg and 16 U/kg) showed considerable functional improvements and goal attainment (9), with no statistical superiority for the higher doses. However, all children were to participate in an individualized, goal oriented HETP, which may have contributed to the significant improvements seen, and which may have also influenced safety outcomes (e.g., a home strengthening program may have mitigated any mild muscle weakening post-injection). Moreover, children were randomly assigned to dose groups and investigators remained blinded to dose throughout the study. Thus, the design does not reflect clinical practice in which addressing a child's functional needs necessitates knowledge of the dose being injected into the muscles in order to appropriately tailor the treatment accordingly.

Of note, the dose ranges per muscle proven to be effective in this study in the 8 U/kg and 16 U/kg groups are below or at the low end of recommended dose ranges based on prior expert opinion. For example, whereas the international consensus statement published by Fehlings and colleagues in 2010 recommended an aboBoNT-A dose range of 5–10 U/kg for the brachialis (10), mean brachialis doses were 2.75–2.92 U/kg in the 8 U/kg group and 5.30–5.38 U/kg in the 16 U/kg group and the maximum brachialis dose used in this study was 6 U/kg. The 2010 consensus paper also recommended a dose range of 5–10 U/kg for the brachioradialis, flexor carpi ulnaris and flexor carpi radialis whereas, again, our study protocol used lower dosing in these key muscles which were often part of the PTMG. Indeed, the maximum doses used at any point for the brachioradialis and/or flexor carpi ulnaris was 3 U/kg and the highest dose used for the flexor carpi radialis was 4 U/kg. Given the efficacy of the tested doses (9), our findings suggest that such recommendations have the potential to lead to overdosing in the different muscles; for example, we note that the 2010 dosing recommendations per muscle are well above those recommended in abobotulinumtoxinA product labeling (17, 18). Our study also suggests that it is possible to inject more muscles in one treatment session, contrary to the restricted number of muscles suggested by some early publications (11), but in line with the recommendations that have been made by other experienced clinicians (19). The dosing used in additional (i.e., non-PTMG) muscles in our study was also significantly lower than in the 2010 guidelines, but this may have been influenced by our study protocol requiring clinicians to deliver a specified volume to the PTMG before deciding on any additional muscles.

In line with the clinical presentation and need for a whole child treatment approach, once lower limb treatment with aboBoNT-A was permitted from Treatment Cycle 2 onwards, a considerable proportion of children also received treatment for their lower limb spasticity. Indeed, the fact that the majority of children received concomitant treatment in the lower limbs is consistent with the fact that the majority of children also had some degree of concomitant lower limb muscle spasticity (76.9% had unilateral and 21.6% had bilateral CP). Concomitant treatment of any other limb in addition to the study upper limb did not appear to influence the frequency or severity of any TEAE (including muscular weakness) reported. Although not the objective of this study, the range of dosing required in the upper limbs appears to offer potential for some level of concomitant dosing of the upper and lower limbs, and thus could facilitate a holistic treatment approach. A previous aboBoNT-A study focusing on lower limb spasticity also illustrated the need for concurrent treatment of upper and lower limb with 10% of children receiving lower limb treatment also receiving upper limb injections when permitted by the protocol (15). The recommended aboBoNT-A dosing for the lower limbs is currently 10 or 15 U/kg/leg (17, 18), and dosing guidelines for lower limb muscle are also available. Nevertheless, decisions on how to distribute the total dose will depend on the clinician's judgement of each individual patient's presentation as well as the prioritization of treatment goals.

Despite the limitations in our protocol for evaluation of dosing in the clinical setting, which included blinded allocation of dose and the fixed requirements associated with injections into the PTMG, our data highlight the many permutations of muscle and dose combinations that can be used to treat upper and lower limb pediatric spasticity with aboBoNT-A. Injections of the upper limb require careful muscle selection and our study allowed flexibility in the upper limb muscles to be injected. However, other parameters may also limit the full generalizability of data to daily practice. One such parameter is the mandated injection volume of 1.6 mL into the study limb for all patients, which is considered a relatively small volume for multi-muscle injections, despite this volume providing benefits for muscle tone, function and achievement of goals (9). In addition, Fehlings et al. recommended that the suggested volume should vary, with smaller volumes for injections intended to improve active function and larger volumes for older children or children with goals aiming to improve appearance, tolerance of orthoses, or facilitation of care (10). Another point to highlight is that all injections in our study were administered using injection guidance for accurate targeting (in contrast to some older studies). The importance of guidance techniques continues to be an important topic in the use of BoNT-A, with more and more centers gaining access to tools such as ultrasound and electrical stimulation.

In summary, this study provides important information for clinicians regarding the muscles and doses used to treat upper (and, in many cases, upper and lower) limb spasticity in children with CP, within a well characterized safety profile. Further work could focus on prospective or retrospective observational studies, which could bring to light further insights into the optimal management of children with CP.

## Data Availability Statement

The original contributions generated for the study are included in the article/Supplementary Material, further inquiries can be directed to the corresponding authors.

## Ethics Statement

Institutional review boards at the participating sites approved the protocol, and the trial was executed in accordance with the Declaration of Helsinki and International Conference on Harmonization Good Clinical Practice Guidelines. A complete list of the institutional review boards is available in the supplementary material. Written informed consent to participate in this study was provided by the participants' legal guardian/next of kin.

## Author Contributions

All authors were involved in data review, analysis and contributed to the interpretation of results, and approved the final version of the article.

## Funding

This work was funded by Ipsen.

## Conflict of Interest

This study was funded by Ipsen. The authors employed or contracted by Ipsen SP, BR, and CT were involved in interpretation of the data; and in review, approval of, and decision to submit the manuscript. The funder had no other role in study conduct or preparation of this report. JO, AT, JC, ND, MB, ED, and MD were investigators in Ipsen-sponsored clinical trials and they or their institutions have received payment for participation. In addition, JO reports consultancy fees for Ipsen and Allergan. AT reports research support and educational grants from Ipsen and personal fees for consultancy from Ipsen. JC reports personal fees for consultancy and speaking from Ipsen. ND reports research support from Ipsen, Allergan, and Merz and personal fees for consultancy and speaking from Ipsen and Allergan. MB reports research support from Ipsen, Allergan, and Merz and personal fees for consultancy and speaking from Ipsen and Allergan. ED reports personal fees from Ipsen and Allergan for speaking, Solstice Neurosciences for consultancy and serves on a US speaker bureau. MD reports personal fees from Ipsen, Allergan and Kashiv Pharma for consultancy.

## Publisher's Note

All claims expressed in this article are solely those of the authors and do not necessarily represent those of their affiliated organizations, or those of the publisher, the editors and the reviewers. Any product that may be evaluated in this article, or claim that may be made by its manufacturer, is not guaranteed or endorsed by the publisher.
