# Supplementary material for: Muscle Selection and Dosing in a Phase 3, Pivotal Study of AbobotulinumtoxinA Injection in Upper Limb Muscles in Children With Cerebral Palsy
Source: Front Neurol. 2021 Oct 29;12:728615. doi: 10.3389/fneur.2021.728615 (PMC8603760; doi:10.3389/fneur.2021.728615)
Supplement: Supplementary file 1 [file Table_1.docx]

| **Belgium** | Comité d’Ethique hospitalo-facultaire, Cliniques Universitaires Saint-Luc. Avenue Hippocrate 10, BE - 1200 Bruxelles. |
| --- | --- |
| **Poland** | Ethics Committee of the Regional Medical Council in Warsaw. 18 Pulawska Street, room 110, 02-512 Warsaw. |
| **Mexico** | “Comite de la Clinica Bajío CLINBA, S.C.” Valenciana # 7 Colonia Paxtitlan. Z.C. 36090 Guanajuato, Gto. |
| **Turkey** | Kocaeli University Clinical Trials Ethics Committee, Kocaeli Üniversitesi Tıp Fakültesi Klinik  Araştımalar birimi Umuttepe Yerleşkesi, 41380, Kocaeli |
| **Israel** | Helsinki Committee Sheba Medical Center, Derech Sheba 2, Tel Hashomer, Ramat Gan 5266202.  Helsinki Committee Soroka University, Medical Center, Rager Boulevard, POB  151 Beer-Sheva 8410101.  Helsinki Committee, Rabin Medical Center, Petach Tikva 4941492.  Helsinki Committee, Tel-Aviv (Sourasky) Medical Center, 6 Weizmann St Tel-Aviv 6423906.  Helsinki Committee, Shaare-Zedek Medical Center, 12 Shmuel Bait St. Jerusalem 9103102. |
| **Czech Republic** | Ethics Committee for Multi-Centric Clinical Trial of the University Hospital Motol, Vuvalu 84, Prague 5, 150 06.  Ethics Committee, St. Anne´s University Hospital in Brno. Pekarska 53, Brno, 65691. |
| **Spain** | Drug Research Ethics Committee (DREC), Agencia de Ensayos Clínicos. Hospital Universitari Vall d'Hebron, Edificio Institut de la Recerca, 3ª planta. Passeig Vall d'Hebrón 119-129, 08035 Barcelona |
| **USA** | University of Texas Southwestern Medical Center, Institutional Review Board. 5323 Harry Hines Boulevard Dallas, Texas 75390-8843 Room C1.206.  Western Institutional Review Board®. 1019 39th Avenue SE Suite 120 Puyallup, WA 98374-2115.  Louisiana State University Health Sciences Center-New Orleans Institutional Review Board. 433 Bolivar Street, New Orleans, LA 70112.  Seton Institutional Review Board, 1400 North IH-35, Suite C3.400. Austin, Texas 78701.  Oregon Health & Science University IRB. 3181 SW Jackson Park Road, Portland, OR 97239.  Beaumont Health System Research Institute. 3811 West Thirteen Mile Road Royal Oak, Michigan 48073-6769.  Columbia University IRB. 154 Haven Avenue, 1st Floor. New York, NY 10032.  Chesapeake Research Review LLC. 6940 Columbia Gateway Drive, Suite 110.  Columbia, MD 21046  Nationwide Children’s Hospital IRB. 700 Children’s Drive. Columbus, OH 43205.  University of Minnesota. 420 Delaware Street SE, D528 Mayo Memorial Building. Minneapolis MN 55455. |
