# Supplementary material for: Muscle Selection and Dosing in a Phase 3, Pivotal Study of AbobotulinumtoxinA Injection in Upper Limb Muscles in Children With Cerebral Palsy
Source: Front Neurol. 2021 Oct 29;12:728615. doi: 10.3389/fneur.2021.728615 (PMC8603760; doi:10.3389/fneur.2021.728615)
Supplement: Supplementary file 2 [file Data_Sheet_1.PDF]

Supplementary appendix 1

Table e1. Maximum Injected Volumes, Dose per Muscle and Number of Injection sites in the Study Upper Limb

**Muscle Group**	**Injection Volume (mL)**	**Number of Injection Sites**	**Dose per Muscle in U/kg (Maximum U)**		
			**Control Group**	**Treatment Groups**	
			**AboBoNT-A 2 U/kg**	**AboBoNT-A 8 U/kg [a]**	**AboBoNT-A 16 U/kg [a]**
**Elbow Flexors**					
Brachialis	0.6 [b]	2	0.75 U/kg (30 U)	3 U/kg (120 U)	6 U/kg (240 U)
Brachioradialis	0.3	1	0.375 U/kg (15 U)	1.5 U/kg (60 U)	3 U/kg (120 U)
**Wrist Flexors**					
Flexor carpi radialis	0.4	1 to 2	0.5 U/kg (20 U)	2 U/kg (80 U)	4 U/kg (160 U)
Flexor carpi ulnaris	0.3	1	0.375 U/kg (15 U)	1.5 U/kg (60 U)	3 U/kg (120 U)
**Additional Muscles**					
Biceps (optional muscle)	0.6 [b]	2	0.75 U/kg (30 U)	3 U/kg (120 U)	6 U/kg (240 U)
Pronator teres	0.2	1	0.25 U/kg (10 U)	1 U/kg (40 U)	2 U/kg (80 U)
Pronator quadratus	0.1	1	0.125 U/kg (5 U)	0.5 U/kg (20 U)	1 U/kg (40 U)
Flexor digitorum profundus	0.2	1	0.25 U/kg (10 U)	1 U/kg (40 U)	2 U/kg (80 U)
Flexor digitorum superficialis	0.3	2 to 4	0.375 U/kg (15 U)	1.5 U/kg (60 U)	3 U/kg (120 U)
Flexor pollicis longus	0.2	1	0.25 U/kg (10 U)	1 U/kg (40 U)	2 U/kg (80 U)
Flexor pollicis brevis/ opponens pollicis	0.1	1	0.125 U/kg (5 U)	0.5 U/kg (20 U)	1 U/kg (40 U)
Adductor pollicis	0.1	1	0.125 U/kg (5 U)	0.5 U/kg (20 U)	1 U/kg (40 U)
Pectoralis major	0.5	1 to 2	0.625 U/kg (25 U)	2.5 U/kg (100 U)	5 U/kg (200 U)
Pectoralis minor	0.5	1 to 2	0.625 U/kg (25 U)	2.5 U/kg (100 U)	5 U/kg (200 U)
**Total dose for the study upper limb**	**1.6**		**2 U/kg (80 U)**	**8 U/kg (320 U)**	**16 U/kg (640 U)**

U=units

a The total dose for the study limb could not exceed 320 U in the 8 U/kg group and 640 U in the 16 U/kg group.

b Dose was administered across the two injection sites.
