# Supplementary material for: Muscle Selection and Dosing in a Phase 3, Pivotal Study of AbobotulinumtoxinA Injection in Upper Limb Muscles in Children With Cerebral Palsy
Source: Front Neurol. 2021 Oct 29;12:728615. doi: 10.3389/fneur.2021.728615 (PMC8603760; doi:10.3389/fneur.2021.728615)
Supplement: Supplementary file 3 [file Data_Sheet_2.pdf]

Looking at muscle selection and the dose of aboBoNT-A injections used for the treatment of pediatric upper limb spasticity

Study number: NCT02106351 **Date of summary:** June 2021
Study start date: April 2014 **Study end date:** September 2018

Please note that this summary only contains information from the full scientific article

The full title of this article is: Muscle selection and dosing in a phase 3, pivotal study of abobotulinumtoxinA injection in upper-limb muscles in children with cerebral palsy

The purpose of this plain language summary is to help you to understand the findings from recent research.

AbobotulinumtoxinA (aboBoNT-A) is approved to treat the condition under study that is discussed in this summary.

The results of this study may differ from those of other studies. Researchers should make treatment decisions based on all available evidence and not on the results of a single study.

1 What was this study about?

- **This summary describes how doctors treated children with cerebral palsy (CP for short) and pediatric upper-limb spasticity (PUL spasticity for short) using aboBoNT-A injections.**
- **In this study, researchers looked at the doses of aboBoNT-A that doctors used and the muscles they injected.**

What is PUL spasticity?

- CP is a condition that affects people's movement and coordination. Many children living with CP have restricted use of their upper limbs (shoulder, elbow, wrist, and hand). This is called PUL spasticity and is caused by involuntary muscle tightness.

How is PUL spasticity treated?

- Botulinum is a toxin produced by a type of bacteria. It causes muscle weakness by disrupting the nerves that control muscles.
- Current guidelines for treating PUL spasticity recommend injecting a medicine containing botulinum toxin into the upper-limb muscles to ease tightness.
- In the Dysport in PUL spasticity study, researchers showed that injections of a medicine containing botulinum toxin, called aboBoNT-A, improved arm movement in children with CP.
- Treatment was most effective at doses of 8 or 16 units of aboBoNT-A per kilogram of body weight (U/kg).
- However, little guidance exists for doctors on choosing the best dose of aboBoNT-A, or on which muscles they should inject when treating PUL spasticity.

What did the researchers want to find out?

- Researchers wanted to better understand what dose of aboBoNT-A to use and which muscles to inject to optimally treat PUL spasticity in children with CP.
- This study uses data from the Dysport in PUL spasticity study, which included 4 treatment cycles.
  - In Cycle 1, children received either 2 U/kg, 8 U/kg, or 16 U/kg of aboBoNT-A.
    - Doctors injected aboBoNT-A into specific muscles in the children's elbows and wrists, but could also inject other upper-limb muscles as needed.
  - In Cycles 2-4, children received aboBoNT-A at doses of either 8 U/kg or 16 U/kg.
    - Doctors could choose different injection sites from Cycle 1. These sites could include muscles in the upper and lower body.
- The researchers looked at which muscles doctors injected with aboBoNT-A. They also studied what doses were given and compared any side effects children experienced.
  - A side effect is something expected or unexpected that may be caused by a medicine or treatment.

2 Who took part in this study?

- Children aged 2-17 years old who had CP and PUL spasticity were included in the study. Overall, 210 children received aboBoNT-A injections.

3 What were the results of this study?

- Across all 4 treatment cycles, the most common upper-limb muscles chosen for injection were in the elbows and wrists, but other upper-limb muscles were also used.
- In Cycles 2-4 over half the children received injections in their lower limbs in addition to other muscles in their upper limbs.
  - The lower-limb muscles most often chosen for injection were at the back of the leg and in the groin.
- Overall, the number of children with side effects was low.
  - The most common side effect thought to be related to injection of aboBoNT-A was unwanted muscle weakness. This occurred in fewer than 1 in 10 children during the first 3 cycles of treatment.
- Side effects did not increase over treatment cycles, at higher doses, or when aboBoNT-A was injected into multiple limbs.

4 What were the main conclusions of this study?

- This study shows that while most children received injections of aboBoNT-A in the muscles of the elbows and wrists, doctors chose a wide variety of other muscles for injection to meet individual children's needs.
- The results also suggest that aboBoNT-A may be effective at lower doses than those currently recommended in treatment guidelines.
- Overall, these results may help doctors to understand how best to use aboBoNT-A to help children reach their individual treatment goals.

5 Who sponsored this study?

This study was sponsored by Ipsen.

6 Further information

- For more information on this study, please visit: **https://clinicaltrials.gov/ct2/show/NCT02106351**
- For more information on clinical studies in general, please visit: **https://www.clinicaltrials.gov/ct2/about-studies/learn**
- If you have any questions about this study, please contact the sponsor, Ipsen, at: **www.ipsenmedicalinformation.com**

We thank all of the patients who took part in this study. Without their support, advances in treatments for medical conditions would not be possible.

Summary prepared by Jake Evans, PhD, at Envision Pharma Group.
Plain language services were funded by Ipsen.
